# Efficacy of 5% Aminolaevulinic Acid and Red Light on *Enterococcus faecalis* in Infected Root Canals

**DOI:** 10.3390/gels9020125

**Published:** 2023-02-02

**Authors:** Teocrito Carlesi, Tatiane Cristina Dotta, Tania Vanessa Pierfelice, Emira D’Amico, Stefania Lepore, Domenico Tripodi, Adriano Piattelli, Simonetta D’Ercole, Morena Petrini

**Affiliations:** 1 Department of Medical, Oral and Biotechnological Sciences, University of Chieti-Pescara, 66100 Chieti, Italy; 2 Department of Dental Materials and Prosthodontics, School of Dentistry of Ribeirão Preto, University of São Paulo, Sao Paulo 14040-904, Brazil; 3 School of Dentistry, Saint Camillus International University for Health Sciences (Unicamillus), 00131 Rome, Italy; 4 Facultad de Medicina, UCAM Universidad Catolica San Antonio de Murcia, Av. de los Jerónimos, 135, Guadalupe de Maciascoque, 30107 Murcia, Spain

**Keywords:** 5-aminolevulinic acid, endodontics, photodynamic therapy, red light, *Enterococcus faecalis*

## Abstract

Background: In this ex vivo study, the aim was to evaluate the effects of ALAD and red light on *Enterococcus faecalis* in infected root canals using a special intracanal fiber. Methods: A total of 70 extracted, single-rooted teeth were used. The teeth were decoronated at the length of the roots to approximately 15 mm and then instrumented. The apical foramen was sealed by composite resin, and the root canals were infected with a pure culture of *E. faecalis* ATCC 29212 for eight days at 37 °C. Following the contamination period, the roots were divided into seven groups, including the positive and negative control groups, and treated as follows: ALAD 45 min; red light activation 7 min; ALAD 45 min and red-light activation 7 min; sodium hypochlorite 2.5% 15 min; sodium hypochlorite 1% 15 min. The samples were taken by three sterile paper points, transferred to tubes containing 1 mL of PBS, and immediately processed for the number of colony-forming units and the cell viability by using live/dead. Results: The best treatment is obtained with 2.5% NaOCl. Except for ALAD + red light vs. 1% NaOCl, a statistically significant difference is recorded for all treatments. The combination of 2.5% NaOCl and ALAD + 7 min irradiation produces an evident killing effect on the *E. faecalis* cells. On the other hand, 1% NaOCl is ineffective for the viability action, with 25% of dead cells stained in red. Conclusions: This ex vivo study shows that ALAD gel with light irradiation is an efficacious protocol that exerts a potent antibacterial activity against *E. faecalis* in infected root canals.

## 1. Introduction

Endodontic therapy is an alternative to tooth extraction and isa complex dental treatment that permits the preservation of the natural tooth in the case of necrosis of the pulp or irreversible pulpitis [1]. Numerous factors are essential to achieve the goal of the elimination of bacterial load and infected necrotic tissue. These include a correct mechanical preparation of the canals, the chemical disinfection by means of irrigants, and the three-dimensional obturation of the endodontic space, aiming at [2]. Eliminating bacterial microorganisms, toxins, and the decontamination of the endodontic area is a crucial moment for long-term success [3]. The success of treatment increases in the case of vital teeth with less infection than necrotic teeth with a higher bacterial load [4].

The most widespread procedures aiming to eliminate intracanal infection include mechanical instrumentation for cleaning and shaping the root canal systems with manual or rotating endodontic instruments (files); the use of irrigating solutions with antimicrobial action, such as chlorhexidine, sodium hypochlorite, and EDTA acid; and application of intracanal medication, such as calcium hydroxide [5]. Although most of the infection is removed by mechanical instrumentation, unfortunately, total elimination of the bacterial infection is not possible within the root canals, even when there is extensive irrigation of sodium hypochlorite (NaOCl) [6]. According to Nunes et al. [1], this is related to the fact that irrigation instruments and solutions are often unable to act in deep areas of root canal systems due to anatomical complexity, in addition to the fact that some bacteria develop resistance to these methods. In addition, several types of bacteria can lodge in the root canal and areas of difficult access to disinfection, such as lateral canals, leading to failure of endodontic therapy [2].

Some authors histologically detected the presence of bacterial biofilms present in the isthmus and accessory canals in the last apical 3 mm of teeth extracted at the end of the treatment of root canal [7]. Other authors show that bacterial infiltration can penetrate the dentinal tubules up to 1000 μm. [8] The anatomically complex endodontic system, with branches, isthmuses, and dentinal tubules, makes it impossible to eliminate the bacterial load with chemo-mechanical instruments only [1].

Typically, Gram-negative anaerobic bacteria predominate among the microbial species present in primary endodontic infections, while Gram-positive facultative aerobic bacteria are linked to secondary endodontic infections [1]. *Enterococcus faecalis* is a facultative Gram-positive bacterium commonly found in roots already treated with infection persistence, and rarely found in untreated root canals [9]. This bacterium is difficult to eradicate as it can survive in extreme environments, with few nutrients, little oxygen, and large variations in temperature and pH [10,11].

In addition to *E. faecalis*, other microorganisms have also been found endodontically including *Porphyromonas endodontalis*, *Bacteroides* spp., *Dialister invisus*, *Fusobacterium nucleatum*, *Treponema denticola*, *Peptostreptococcus* spp., and *Veillonella* spp. [12]. However, Nunes et al. [1] concluded that Enterococcus faecalis is root canals’ most prevalent microbial species before disinfection.

Since the persistence of bacteria in the endodontic root canal system causes medium- and long-term failure, several light-based approaches have been proposed to increase the antibacterial action. Indeed, the additional use of light irradiation at specific wavelengths could act synergically with chemical irrigants to increase the antibacterial activity. Petrini et al. and D’Ercole et al. show that 880 nm light irradiation increases the effects of NaOCl and chlorhexidine at low concentration synergically, permitting the eradication of *E. faecalis* and *Pseudomonas aeruginosa*, with results comparable to the single antimicrobials at higher concentration. Surprisingly, the antibacterial effect was also confirmed after 1 week following the last irradiation [13,14,15].

However, the efficacy of photoinactivation is dependent on the presence of endogenous photosensitizers inside bacteria, and this is a consequence of other factors, such as the culture conditions [3,13,14,15]. Therefore, photodynamic therapy consists of the prior application of an exogenous photosensitizer that selectively accumulates in pathological tissues. The activation of these substances by a specific light with an appropriate wavelength generates free radicals and singlet oxygen, which are cytotoxic, causing the eradication of bacteria, or neoplastic cells, in which the photosensitizer was accumulated [16,17].

The amino acid 5-aminolaevulinic acid (ALA) is the precursor of a photosensitizer, as it is converted in situ into protoporphyrin IX (PpIX), a molecule that is part of the reaction of formation of the heme group [18,19]. ALA and its derivatives have been used with excellent results not only for their antibacterial properties but also for cancerous and precancerous therapies in dermatology, and in oral cavity diseases [20]. The photoactivation of PpIX is given by a red LED light with a wavelength of 630 ± 10 nm; this causes direct and indirect antibacterial reactions, in the presence of oxygen, in bacterial cells. The “Aladent” gel (ALAD), a registered trademark covered by a patent, contains 5% aminolaevulinic acid, which has antibacterial properties even in the absence of LED light, due to the presence of potassium sorbate and benzoate sodium [19].

ALAD is effective against Gram-positive and negative bacteria, such as *Staphylococcus aureus*, *E. faecalis*, *Escherichia coli*, *Veillonella parvula*, *Porphyromonas gingivalis*, and *P. aeruginosa* [19]. Furthermore, during in vitro studies, a solution containing 50% of ALAD in addition to photoactivation of 7 min of red LED light can have a total inactivation and effective lethal (95% of cell death) towards *E. faecalis* [19,21].

There is only a case report about the application of ALAD activated by red LED in endodontics that showed the promising clinical applications of this protocol [18]. Therefore, the aim of this ex vivo study was to verify the effects of ALAD and red light on *Enterococcus faecalis*, in infected root canals, using a special intracanal fiber to improve the effectiveness of the treatment.

## 2. Results and Discussion

**Figure 1:**
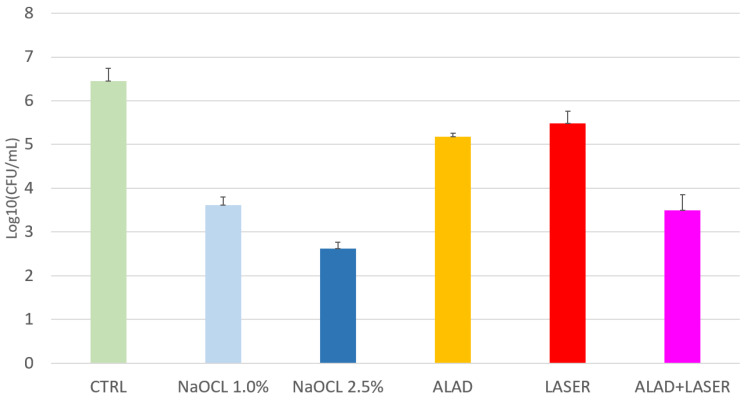
Cultivable *E. faecalis* cells count (log_10_CFU/mL) after several treatments. Data are presented as mean ± standard deviation (SD) and are expressed relative to CTRL (unexposed bacteria). LSD shows that, except for ALAD + red light vs. 1% NaOCl, a statistically significant difference is recorded for all treatments. * *p* < 0.05.

The colony-forming unit values are displayed in Figure 1. ANOVA analysis shows significant differences with *p* < 0.001 among the groups.

The LSD test shows that all treatments significantly decrease (*p* < 0.05) the CFUs, in respect to the positive controls (6.459 ± 0.289 log_10_CFU/mL).

NaOCl shows the higher antibacterial activity demonstrated at both concentrations: 2.5% (2.627 ± 0.145), and at 1% (3.617 ± 0.179).

The laser irradiation alone induces a slight decrease in CFUs: 5.488 ± 0.276 log_10_CFU/mL.

ALAD alone decreases the bacterial load to 5.178 ± 0.086 log_10_CFU/mL. However, a significant higher antibacterial activity is shown by ALAD + PDT (3.495 ± 0.365 log_10_CFU/mL).

Figure 1 shows the effect of different treatments on viable and culturable *E. faecalis* ATCC 29212.

All the treatments significantly reduce the log CFU in respect to the control.

The best treatment is obtained with 2.5% NaOCl, with statistically significant differences in respect to all the treatments. Except for ALAD + red light vs. 1% NaOCl, a statistically significant difference is recorded for all treatments.

**Figure 2:**
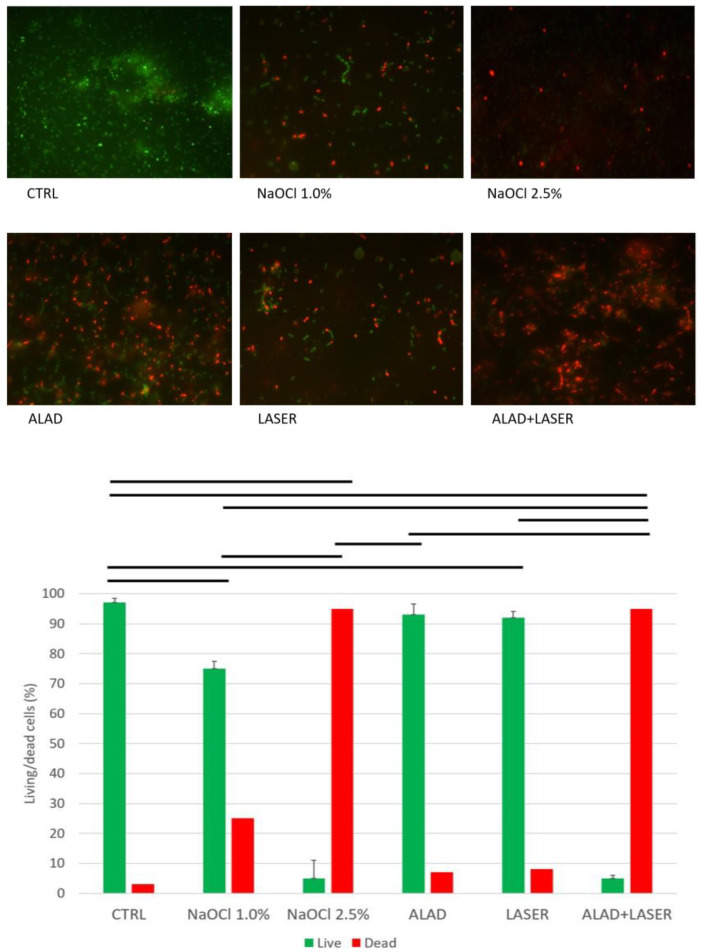
Representative live/dead images of untreated (CTRL) and treated Enterococcus faecalis. The histogram shows the percentages of viable (green bars) and dead (red bars) cells. The horizontal bars show the presence of statistically significant differences (*p* < 0.05) between the groups.

These effects are confirmed by live/dead observation (Figure 2).

The more significant effect is visible for the treatments 2.5% NaOCl and ALAD + 7 min irradiation. They produce an evident killing effect on the *E. faecalis* cells, characterized by the presence of 5% of viable cells stained in green.

The treatment of NaOCl alone at a lower concentration (1%) is ineffective for viability, with 25% of dead cells stained in red. ALAD and red light alone do not produce a relevant effect on the viable cells in respect to the control.

The application of novel technologies for the instrumentation and the three-dimensional obturation of endodontic canals, endodontic failures, or relapses, especially in teeth with complex anatomy, are still a significant concern in dentistry. Therefore, many techniques to increase the antimicrobial action of chemical irrigants have been described. In particular, light has been used not only to exploit its thermal properties but also to favor a greater penetration of the irrigants into the lateral channels, thanks to the turbulence on the liquids exerted by the photons [22].

In this ex vivo study, the efficacy of photodynamic therapy mediated by the simultaneous use of a novel aminolaevulinic-acid-containing product (ALAD) and red irradiation was tested against biofilm *E. faecalis*, one of the most prevalent microbial species in root canals in the presence of endodontic failures and secondary infections.

The ex vivo model of root canals infected with bacteria is closer to in vivo conditions for antimicrobial evaluations of different treatments [23].

ALAD gel contains potassium sorbate and sodium benzoate as preservatives, which could contribute to the bactericidal effect [19]. Therefore, the results demonstrate that the association of ALAD gel and red light for 7 min has greater efficacy in CFUs and cell viability compared to the two single treatments LASER and ALAD alone. A narrative review and case report by D’Ercole et al. [18] demonstrates a total decrease in CFU/mL after 45 min of incubation with ALAD gel and subsequent 7 min of LED irradiation, compared to samples with only incubation with Aladent (ALAD) (210 CFU/mL). Furthermore, in the study by Radunovic et al. [19], total inactivation of *E. faecalis* (95% of dead cells) was possible with a solution containing 50% ALAD applied for 45 min in combination with light at 630 nm for 7 min.

It is essential to highlight that although there is use of a laser device, a source of irradiation, a very low intensity of light, 50 mW, has been applied. This intensity value is significantly lower than the values used in previous studies on photodynamic therapy and endodontics, in which the light intensity values were higher, up to 1 W [15,19,21]. As shown by the slight effects of LASER irradiation on bacterial CFUs, the significant microbial decrease obtained in this study results from the synergic effect of ALAD + light. This protocol provides a drop of about 3 log reduction in respect to the positive controls, so this antibacterial effect could be considered particularly effective if we believe that a log reduction of 1 is equivalent to a 10-fold decrease corresponding to 90% of bacterial reduction.

The antibacterial effects of ALAD gel followed by red irradiation are well-known, but in this study, this association was tested for the first time on bacterial biofilm growth on root canals rather than in planktonic bacteria [18,19,21].

Furthermore, in terms of CFUs, the results obtained are perfectly superimposable to those obtained with 1% NaOCl. A lower efficacy is reported compared to 2.5% NaOCl against a biofilm structure acquired with more complex morphology. The live/dead analysis shows that the more significant effect of killing is visible for the treatments 2.5% NaOCl and ALAD + 7 min irradiation and that the treatment of 1% NaOCl is ineffective for the viability action.

However, it is important to underline that the use of high concentrations of NaOCl are not advisable for clinical activity because they are cytotoxic to fibroblasts cells, and produce irritation of the periapical tissues with an unfavorable odor and taste [15]. Furthermore, Teixeira et al. [24] demonstrate that NaOCl is able to maintain cell viability only with the 0.01% dilution, and Simbula et al. [25] show that higher concentrations are related to lower percentages of cell viability.

Some authors proposed the use of photodynamic therapy in the primary endodontic treatment of teeth with periapical lesions [26,27] and in teeth already treated endodontically [28,29].

Based on previous studies regarding the bactericidal action of ALAD gel and red light against *E. faecalis,* and the tests performed with a LED lamp (Fotosan) equipped with fiber and toluidine blue in photodynamic therapy in endodontics, some authors proposed a fiber system connected to the light to optimize the application of light inside the root canals [18,30].

During the experiments, the fiber tip light was inserted inside the root canals up to the end of these, and throughout the irradiation inside the canals, slow movements were performed inside the root canals to reach the entire surface of the biofilm.

The results of this study are in agreement with others on the efficacy of endodontic photodynamic therapy, confirming that this treatment has efficacy in reducing the bacterial load in root canals [1,31]. The photosensitizing agents most used in endodontics are phenothiazine derivatives, methylene blue, and toluidine, activated with different types of lamps (LEDs and lasers) with different wavelengths, with or without dedicated intracanal fiber [1,32]. They are synthetic dyes with several disadvantages, such as tissue staining, toxicity, poor efficacy on biofilm, and poor selectivity [33].

ALAD gel has the advantage of being an organic compound, free of side effects, and the formula, due to some preservative substances, also has an acid pH of 3.50, and these aspects would confer greater effectiveness against Gram-negative bacteria compared to other formulas based on aminolaevulinic acid [19,34].

According to D’Ercole et al. [18], photodynamic therapy has become a promising antimicrobial strategy to aid in endodontic treatment, with easy and quick application that can be used in single or several sessions, in addition to not allowing forms of microbial resistance.

## 3. Conclusions

In conclusion, this ex vivo study shows that ALAD gel with light irradiation is an efficacious protocol that exerts a potent antibacterial activity against *E. faecalis* in infected root canals. The efficacy of this treatment could open new opportunities for root canal disinfection during endodontic treatment.

## 4. Materials and Methods

### 4.1. Sample Preparation

A total of 70 teeth, stored in physiologic saline, were used. The inclusion criteria for teeth choice were: humans extracted single-rooted teeth, without root fractures or prior endodontic treatment without the endodontic space obstructed by calcifications or other materials.

The use of extracted teeth for the in vitro study was previously authorized by the Ethical Committee of the University of Chieti-Pescara (reference number: BONEISTO N. 22 10.07.2021). The teeth were decoronated with the use of diamond burns. The length of the roots was then standardized to approximately 15 mm. NiTi Mtwo instruments were used in a 16:1 handpiece (Anthogyr, Sallanches, France) in conjunction with a high torque endodontic electric motor (E-Go, Sweden and Martina, Padova, Italy) at 150 rpm in a simultaneous technique. Working length was established by passing a size 10 K-file (Dentsply-Sirona Endodontics, Ballaigues, Switzerland) in the canal until visible at the apex and subtracting 1 mm. Instrumentation was continued until Maximal Apical Foramen 30 (taper 0.05). During shaping, each canal was irrigated between each successive instrument with 2.5 mL of 5.25% NaOCl using an endodontic syringe (Navi tip, Ultradent, South Jordan, UT, USA). After root canal instrumentation, the smear layer was removed by irrigating the canal with 1 mL of 17% ethylenediamine tetraacetic acid solution (Ogna, Italy) for 2 min, followed by a final flush with 1 mL of 5.25% NaOCl (Ogna, Italy) for 30 s. Then, the canals were washed with 1 mL of 5% sterile saline solution. All root canals were irrigated with a 30-gauge needle (Sigma-Aldrich, Steinheim, Germany).

The apical foramen was sealed by composite resin (P60, 3M ESPE, Seefeld, Germany) and the root surface was waterproofed with coats of clear nail varnish to prevent bacterial leakage. Then, the samples were placed in test tubes, immersed in TSB (Oxoid, Milan, Italy), ultrasonicated for 1 min to release the entrapped air and allow penetration of the culture media into root canal irregularities sterilized by autoclaving.

### 4.2. Strain Culture Condition

*Enterococcus faecalis* ATCC 29212 was used for the study because it is known to resist treatment in root canals and adheres to dentine. A pure culture of *E. faecalis* was grown brain heart infusion broth (BHI, Oxoid, Milan, Italy) and incubated overnight at 37 °C in aerobic condition. A standard inoculum of 10^8^ CFU mL^−1^ concentration, obtained using a spectrophotometer, was injected into the root canal using an endodontic needle for the root canals contamination for 8 days at 37 °C aerobically under gentle shaking and the final biofilm generation. Culture media was replenished every 2 days. Inoculum concentration was confirmed by determining the colony-forming units per milliliter (CFUs mL^−1^) using tenfold serial dilutions.

### 4.3. Aladent

The ALADENT gel (ALAD) (Alphastrumenti Srl, Melzo, Italy) used in this study, contained 5% of 5-delta aminolaevulinic acid and other components, covered by a patent. ALAD is liquid at a temperature less than 28 °C and gels at higher temperatures. The study was performed at room temperature, under a laminar flow hood, and 10 µL of gel was inserted in the root canals of the group exposed after 8 days following the bacterial inoculation and left to incubate at dark for 45 min.

### 4.4. Light Source and Fiber TIP

**Figure 3:**
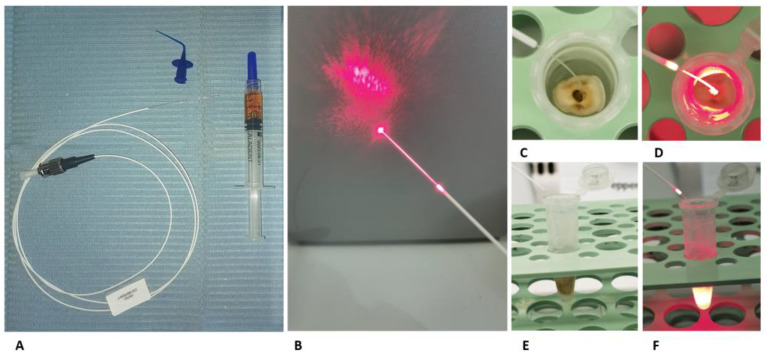
(**A**) The optical fiber used for ALAD-PDT, the tip to deliver the ALAD gel inside the canal roots, and the ALAD syringe. (**B**) The tip of the optical fiber during light irradiation. (**C**,**E**): occlusal and lateral view of the optical fiber inside the root canal, respectively. (**D**,**F**): occlusal and lateral view of the optical fiber inside the root canal during the light irradiation, respectively.

A diode Laser AlGaInP, TL-07 (Alphastrumenti Srl, Melzo, Italy) with 635 nm wavelength was used as a light source. The irradiation was performed for 7 min in continuous emission at 50 mW of intensity by using a OM1 silica multimode fiber, characterized by a diameter of62.5 µm at the core and 125 µm outside (Alphastrumenti, Italy). The fiber end was stripped of the external coating at the distal end for 30 mm, exposing the bare fiber (Figure 3A,B).

During the experiments, the fiber tip light was inserted inside the root canals up to the end of these. During the irradiation inside the canals, slow movements were performed inside the root canals. As shown in our previous studies [13,19,21], the irradiation was performed under a laminar flow hood in the dark under aseptic conditions in all the experiments.

### 4.5. Study Model

Following the contamination period, the 70 roots were divided into 7 groups of 10 roots, including the positive and negative control groups (Figure 3C–F).

In details, the different groups were treated as followed:

- ALAD: samples incubated 45 min with ALAD gel;
- LASER: samples irradiated 7 min with the optical fiber at 50 mW;
- ALAD + LASER: samples incubated 45 min with ALAD and then irradiated 7 min with the optical fiber at 50 mW;
- NaOCl 2.5%: samples incubated 15 min with sodium hypochlorite 2.5%;
- NaOCl 1.0%: samples incubated 15 min with sodium hypochlorite 1%;
- CTRL: positive control, only *Enterococcus faecalis* ATCC 29212, received no treatment;
- Negative control: uninoculated sample.

The irrigation with ALAD and sodium hypochlorite was performed with disposable syringes and 30-gauge NaviTip needles (Ultradent, South Jordan, UT, USA) taken up to 1 mm of the working length. NaOCl was inactivated with 10% sodium thiosulfate for 1 min. The root canal was rinsed with 100 µL of sterile PBS and the samples were taken by the sequential use of 3 sterile paper points placed to the working length. Each paper point remained in the canal for 1 min. Paper points were transferred to tubes containing 1 mL of PBS and immediately processed.

### 4.6. Determination of Colony-Forming Units (CFU)

The effect of different treatments on viable and culturable *E. faecalis* ATCC 29212 was determined by measuring the number of colony-forming units of each sample.

Samples were vortexed for 1 min and 100 µL of selected tenfold serial dilutions were spread on Trypticase Soy agar (TSA) plates (Oxoid, Milan, Italy). After overnight incubation at 37 °C, numbers of surviving bacteria (colony-forming units: CFU) were counted and then transformed into actual counts based on the known dilution factors, and then were expressed as log10 (CFU/mL).

### 4.7. Cell Viability Assay and Fluorescent Microscopy

The cell viability was evaluated thought fluorescent microscopy by using BacLight LIVE/DEAD Viability Kit (Molecular Probes, Invitrogen, Waltham, MA, USA). SYTO 9 stains viable cells with a green, fluorescent signal, and propidium iodide stains cells with impaired membrane activity with a red fluorescent signal.

After treatments, the samples were washed with PBS and stained as indicated by manufacturer. The images observed at fluorescent Leica 4000 DM microscopy (Leica Microsystems, Milan, Italy) were recorded at an emission wavelength of 500 nm for SYTO 9 (green fluorescence) and of 635 nm for propidium iodide (red fluorescence). Two investigators counted the number of living cells (green) and the dead ones (red) on ten different images, so average values (±standard deviation) and the relative percentage of live/dead cells were calculated.

### 4.8. Statistical Analysis

All data were recorded in a Microsoft Excel data sheet (Washington, DC, USA). CFUs were converted in logarithmic forms. Then, SPSS Statistics for Windows, version 21 (IBM SPSS Inc., Chicago, IL, USA) was used to calculate the analysis of variance (ANOVA) and Fisher’s least significant difference (LSD). Statistically significant differences were considered to be a *p*-value < 0.05.

## Data Availability

The data presented in this study are available on request from the corresponding author.
